# Migration Patterns and Meteorological Drivers of the Rice Leaf Roller in Western Hunan Province, China

**DOI:** 10.3390/insects17050466

**Published:** 2026-04-30

**Authors:** Jia-Hao Zhang, Xue-Yan Zhang, Yi-Yang Zhang, Jian Tian, Xiao-Yu Ouyang, Li Yin, Yan Wu, Juan Zeng, Shi-Yan Zhang, Gao Hu

**Affiliations:** 1 State Key Laboratory of Agricultural and Forestry Biosecurity, College of Plant Protection, Nanjing Agricultural University, Nanjing 210095, China; 2 National Agro-Tech Extension and Service Center, Ministry of Agriculture and Rural Affairs of the People’s Republic of China, Beijing 100026, China; 3 Plant Protection and Plant Quarantine Station of Zhijiang Dong Autonomous County, Huaihua 418400, China; 4 Hunan Plant Protection and Plant Quarantine Station, Changsha 411229, China; 5 Guizhou Key Laboratory of Agricultural Biosecurity, Guiyang University, Guiyang 550005, China

**Keywords:** *Cnaphalocrocis medinalis*, trajectory analysis, atmospheric background

## Abstract

The rice leaf roller (RLR), *Cnaphalocrocis medinalis* (Guenée), is a major migratory pest that threatens rice production across East Asia. Effective management of migratory pests relies fundamentally on accurately identifying their source areas, population dynamics, and key environmental drivers. Western Hunan is a critical rice-growing region characterized by unique topography and varied climates, making it a principal pathway for RLR migration. Based on 14-year (2011–2024) monitoring datasets, we identified substantial interannual variability in July RLR abundance in Western Hunan, when the population typically peaks, highlighting the episodic and unstable nature of regional infestations. Back-trajectory simulations reveal that heavy occurrence years of RLR feature clear northward migration pathways from the Indo-China Peninsula and South China to Western Hunan in July, supported by strong southerly winds along the route. Multiple linear regression analysis further shows that spring warmth initially facilitates high population accumulation in source regions, and the synergistic effect of source-region precipitation deficits and abundant local rainfall triggers large-scale immigration into Western Hunan. These meteorological factors collectively account for up to 66% of the interannual variability in RLR population fluctuations, confirming that climatic conditions largely determine outbreak severity. This provides a robust quantitative framework for regional early-warning systems and sustainable pest management in migratory corridors.

## Simple Summary

The rice leaf roller is a small moth that travels long distances by wind and causes serious damage to rice crops in East Asia. Western Hunan, a major rice-growing region, often lies on the main migration path of this pest. We analyzed 14 years of field monitoring data to understand why the number of rice leaf rollers in Western Hunan varies so much from year to year, particularly in July when the population usually peaks. We found that in years with severe infestations, the moths come from the Indo-China Peninsula and southern China, being carried northward by strong southerly winds. Warm spring weather in those source areas helps the insects build up large populations. Later, when these source regions become unusually dry, while Western Hunan receives plenty of local rainfall, huge numbers of moths migrate into the area. These weather patterns account for approximately two-thirds of the year-to-year changes in pest numbers. Understanding this link between weather and insect migration can help researchers forecast outbreaks and support farmers in protecting their rice crops more sustainably, thereby reducing losses and the need for chemical sprays.

## 1. Introduction

The rice leaf roller (RLR), *Cnaphalocrocis medinalis* (Guenée, 1854) (Lepidoptera: Crambidae), is a widely distributed migratory pest affecting rice crops throughout East Asia. It exhibits a migration pattern similar to that of the brown planthopper (BPH), *Nilaparvata lugens* (Stål, 1854) (Hemiptera: Delphacidae) and the white-backed planthopper, *Sogatella furcifera* (Horváth, 1899) (Hemiptera: Delphacidae), all of which migrate over long distances along the East Asian Insect Flyway in Asia [1]. In China, the average annual occurrence area of the rice leaf roller is 1.6 million hectares, with an average annual control area reaching 2.1 million hectares. The most severely affected year was 2007, with an occurrence area of 2.62 million hectares and a control area of 3.97 million hectares. This caused a significant reduction in rice production and seriously endangered China’s food security [2].

The RLR possesses a strong migratory ability and primarily undertakes long-distance flights at night. Each year, it exhibits distinct seasonal north–south round-trip migrations in China, causing severe damage to rice and other crops [3]. As the RLR lacks a diapause mechanism, it cannot overwinter in regions where the mean January temperature is below 4 °C (typically north of 30°N) [1,4,5,6]. Therefore, RLR populations in eastern China rely entirely on the annual arrival of immigrant populations from perennial breeding areas such as Indochina and southern China [1]. Due to its relatively small physical size among insects, the RLR has limited self-propulsion during long-distance migration; consequently, its migratory activities are significantly influenced by weather and climate conditions [7]. Historical outbreaks, such as the 2007 crisis, have demonstrated that a robust southwest monsoon can significantly increase the density of immigrant moths in the Yangtze River Basin, while abnormal local precipitation often induces “forced settling,” leading to rapid population aggregation in specific regions [8]. Beyond seasonal variations, global climate change introduces new complexities by altering regional atmospheric dynamics. Climate-induced changes to the East Asian summer monsoon—particularly the delayed retreat of southerly winds—are disrupting the fall migration patterns of the RLR, preventing southward emigration and creating an ecological trap in the Lower Yangtze River Valley [9]. The increasing frequency of extreme weather events, such as severe heatwaves and the prolonged Meiyu season (a period of persistent precipitation and high humidity during June and July, spanning from the middle and lower reaches of the Yangtze River to southern Japan and Korea) and super typhoons [10,11,12], is profoundly disturbing the spatiotemporal distribution and infestation patterns of insect pests [13]. Among these, extremely high temperatures (exceeding 37 °C) pose a direct physiological threat to RLR, significantly reducing adult longevity and female fecundity, impairing their migratory capacity, and ultimately exacerbating the threat to regional rice production [14,15]. Shifts in precipitation regimes can affect the RLR population by modulating relative humidity (RH), which is a critical factor for the development of the RLR. High humidity (typically > 80%) is essential for egg hatching and the survival of early-instar larvae, as these stages are highly susceptible to desiccation [16].

**Figure 1:**
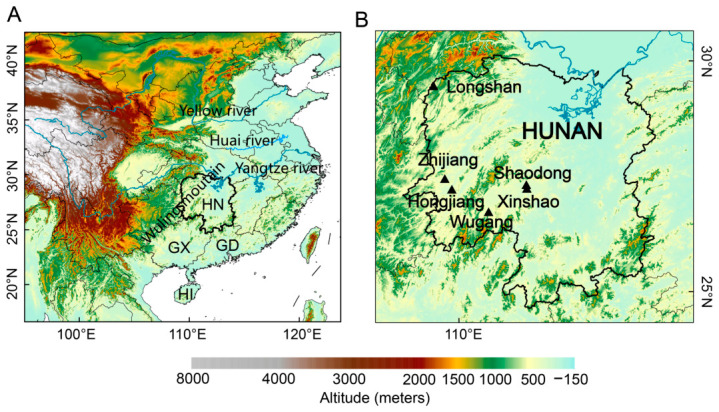
(**A**) Topographic map of the study area, with labels indicating the main provincial names (HN: Hunan, GX: Guangxi, GD: Guangdong, HI: Hainan), rivers and mountains involved. (**B**) Map of Hunan Province. The six triangles indicate the locations of plant protection stations (PPSs) in Western Hunan: Longshan (109.44° E, 29.46° N), Zhijiang (109.68° E, 27.44° N), Hongjiang (110.00° E, 27.21° N), Wugang (110.63° E, 26.73° N), Xinshao (111.48° E, 27.32° N), and Shaodong (111.74° E, 27.26° N).

Hunan Province, situated in the middle and lower reaches of the Yangtze River (Figure 1), is characterized by a subtropical monsoon climate and is recognized as one of China’s largest rice-producing regions. In recent years, its rice planting area has reached approximately 3.95 million hectares, ranking first nationwide in both cultivation area and total yield [17,18]. Favorable climatic conditions and large-scale cultivation, however, contribute to frequent and severe pest outbreaks, with an annual accumulated affected area exceeding 20 million hectares. Migratory pests such as the BPH and RLR account for nearly half of this damage, posing serious threats to rice production [19]. In Hunan, RLR typically migrates into this region in May and completes five to six generations annually. The third, fourth, and fifth generations cause severe damage, with the fourth generation being the most destructive, while the second and sixth generations cause relatively mild damage [20].

The Western Hunan region, situated within the Wuling Mountains, is characterized by a prominent “trough-like” landform. Geographically, the Wuling mountain system extends in a northeast–southwest orientation, forming a natural corridor stretching from Guangxi to Western Hunan. From May to July, this region undergoes rapid warming, with temperatures rising from approximately 21 °C to 28 °C (Supplementary Figure S1). This period coincides with the coming of the rainy season, with June precipitation reaching up to ~290 mm. Furthermore, wind rose analysis reveals that the wind field during this period is characterized by two primary directions: southerly and south-southwesterly, with averaged wind speeds exceeding 4.0 m/s (Supplementary Figure S2). This unique combination of a corridor landform—characterized by the “barrier” and “uplift” effects—and stable southerly airflow renders the region a vital ecological corridor and a high-risk settling zone for migratory pests such as RLR and rice planthoppers [21]. Despite its ecological significance, the specific coupling mechanisms between these regional climatic features and long-term population dynamics remain poorly understood, and targeted studies on RLR in this unique mountainous environment are still limited.

This study focused on the RLR population in Western Hunan from 2011 to 2024. Using fourteen years of monitoring data, trajectory analysis, and meteorological field analysis, we identified the major source regions and examined the meteorological conditions influencing migration. The results aim to clarify the occurrence patterns of RLF in Western Hunan and provide a scientific basis for precise forecasting and effective management of this migratory pest.

## 2. Materials and Methods

### 2.1. Pest Monitoring Data

Five-day interval field survey and daily light trap data for the RLR in Western Hunan from 2011 to 2024 were provided by the National Agro-Tech Extension and Service Center (NATESC). The data cover historical records from six monitoring stations (Figure 1): Zhijiang (109.69° E, 27.45° N; 2011–2024), Hongjiang (109.84° E, 27.21° N; 2011–2024), Xinshao (111.47° E, 27.24° N; 2011–2024), Longshan (109.45° E, 29.46° N; 2012–2024), Wugang (110.64° E, 26.73° N; 2012–2024), and Shaodong (111.46° E, 27.32° N; 2015–2024). The field survey data were collected each morning by local plant protection stations. In representative rice fields, a bamboo pole approximately 1 m long was held horizontally and swept against the wind through rice clusters; the number of RLR adults taking flight was recorded. Based on the area swept, the number of moths that took off was converted into the number of moths per unit area. The light-trap data were collected using ultraviolet lamps to attract adult RLR moths. The traps operated automatically from sunset to sunrise each day. Captured moths were dried for identification, and RLR individuals were collected and counted every morning to monitor their occurrence periods and population density.

### 2.2. Meteorological Data

Two atmospheric reanalysis datasets covering 2011–2024 were used in this study. The daily ERA5 reanalysis data with 0.25° × 0.25° resolution from the European Centre for Medium-Range Weather Forecasts (ECMWF) was employed to analyze meteorological conditions, including daily precipitation, near-surface temperature, and the 900 hPa (≈1000 m above ground) horizontal wind field. The Final Operational Global Analysis (FNL) reanalysis from the U.S. National Centers for Environmental Prediction (NCEP) and the National Center for Atmospheric Research (NCAR) with 1.0° × 1.0° spatial resolution and 6-hourly time intervals was used for trajectory simulation.

### 2.3. Ovary Dissection

From 9 June to 16 September 2024, female RLR moths were collected every three days from the field using insect nets. Following the method described by Zhang et al. [22], we dissected the collected females to determine their ovarian development stages and mating status, which were then used to classify the population source (local, immigration or emigration) for each generation peak recorded in the field surveys. The specific procedure was as follows: During dissection, each moth was placed dorsal side up and fixed in a wax dish containing a thin layer of water; using forceps to hold the body, an insect pin was inserted at the end of the abdomen and carefully cut along the junction between the dorsal and ventral sides to expose the internal organs. Under a stereomicroscope, tissues were separated with insect pins to observe and record the ovarian development stage based on the following criteria: Level 1, ovarioles short and transparent, with oocytes faintly visible after 12 h; Level 2, oocytes formed with 50% milky yolk deposition; Level 3, 5–10 pale-yellow mature eggs with waxy follicular relics at the base; Level 4, ~15 mature eggs occupying half the ovariole length, without relics; and Level 5, atrophied ovarioles with 8–10 remaining eggs (some malformed or fused) and occasional waxy relics at the base. Mating status was determined by examining the presence of spermatophores, and the mating rate (%) was calculated as (number of individuals with spermatophores/number of individuals dissected) × 100%. Based on the above results, population sources were classified into four types: predominantly immigrant type (Level 1 females 0–2%, mating rate > 80%), partly immigrant (Level 1 females 5–10%, mating rate 70–80%), locally reproducing (Level 1 females 11–34%, mating rate 30–69%), and mostly emigrant (Level 1 females > 35%, mating rate < 30%).

### 2.4. Migration Trajectory Simulation

This study employed a three-dimensional trajectory analysis model [23] to simulate and analyze the migration pathways of the RLR. The trajectory model was developed using the FORTRAN language by coupling autonomous flight parameters of RLR into the Weather Research and Forecasting (WRF) model version 4.0 (https://www.mmm.ucar.edu/models/wrf (accessed on 4 February 2026)). The WRF model used FNL reanalysis data as initial and boundary conditions, with terrain data at a spatial resolution of 2′. A single-layer nesting scheme was applied over a simulation area of 130 × 150 grid points, with the center located in Western Hunan. The model output provided hourly meteorological fields at 30 km × 30 km grid spacing, serving as the high-resolution meteorological background field for subsequent trajectory analysis. Backward trajectory simulations were conducted for all six stations as starting points, using flight parameters adopted from previous studies [24,25,26,27,28]. Flight timing was defined based on Beijing Standard Time: take-off was set at 05:00 local time, and landing at 20:00 of the previous day. Trajectories were back-tracked continuously over three consecutive nights, with the endpoint of each night’s trajectory serving as the take-off point for the next night’s simulation. The self-powered flight speed of RLR was set at 0.8 m s^−1^. Four flight altitudes were selected: 500 m, 750 m, 1000 m, and 1250 m above ground level. Furthermore, if the air temperature falls below the low-temperature flight threshold, the insect’s wing-beat frequency decreases or even ceases, ultimately causing it to land in a specific area. In this study, the low-temperature flight threshold for RLR was set at 12.9 °C; the operation of the trajectory simulation would terminate whenever the ambient temperature was lower than this value. Valid trajectory endpoints were screened based on biological and ecological suitability: (i) the endpoint must fall within agricultural areas containing suitable host plants at appropriate growth stages; and (ii) the region must have recorded RLR occurrence capable of serving as a potential source population.

### 2.5. Atmospheric Circulation Background Analysis

Meteorological background fields were analyzed using Python 3.8 (https://www.python.org/downloads/ (accessed on 7 February 2026)). ERA5 datasets were employed to compare meteorological conditions between three typical heavy occurrence years and three typical light occurrence years. The variables extracted included the monthly mean near-surface air temperature (2 m) in May, as well as the 900 hPa horizontal wind field (approximately 1000 m above ground level) and precipitation during early to mid-July. Three-year composite mean fields for both heavy occurrence years and three typical light occurrence years were calculated. Comparison of these composites and their differences allowed us to identify how variations in these meteorological factors influence the RLR migration dynamics and outbreak intensity across different occurrence levels.

### 2.6. Data Preprocessing

To mitigate the non-linear effects caused by orders-of-magnitude differences during outbreak years and to ensure the dataset met the normality assumptions for regression analysis, a logarithmic transformation was applied to the raw counts (N): Y’ = log_10_(N + 1).

In this study, we focus on the interannual variability of RLR abundance and its driving factors. To isolate climate-driven interannual variabilities and remove long-term background trends, linear detrending was applied to both log-transformed RLR abundance data and all meteorological factors. This procedure was intended to eliminate potential confounding factors, including global warming trends, advancements in agricultural pest control technologies, and long-term shifts in regional vegetation cover. For each variable, the residuals from a linear regression against “Year” were all extracted and subsequently standardized. The interannual variation of RLR abundance hereafter refers to the results derived from the standardized detrended time series of RLR abundance.

### 2.7. Multi-Factorial Model Construction

The cumulative population of RLR recorded in Western Hunan during July (2011–2024) served as the dependent variable. To quantitatively analyze the contributions of key meteorological drivers to the annual population dynamics of the RLR in Western Hunan, this study developed a multi-factorial predictive model by multivariate regression analysis combined with hierarchical partitioning.

#### 2.7.1. Selection of Meteorological Factors

Based on the migratory ecology and life cycle of RLR, we constructed an explanatory framework comprising four critical meteorological factors extracted from the ERA5 dataset. The geographic region corresponding to each factor is shown in Supplementary Figure S3.

(1) May Temperature in Source Regions (Spring_T): Represents early-season survival rates and the initial population accumulation potential in the source areas.

(2) July Meridional Wind Component (V_Wind): Characterizes the atmospheric transport potential—south-to-north air currents that facilitate long-distance transport of moth populations from low-latitude source regions to Western Hunan.

(3) Precipitation in Source Regions (Rain_S): Reflects meteorological conditions affecting the “takeoff” phase, where extreme rainfall might wash out or inhibit the emigration of populations.

(4) Local Precipitation in Western Hunan (Rain_L): Serves as the primary trigger for the “forced-settling” (deposition) of migratory populations, leading to regional immigrations.

#### 2.7.2. Statistical Modeling and Contribution Assessment

A quad-factor multiple linear regression (MLR) model was established to evaluate the collective impact of the drivers:

Y = *β*_0_ + *β*_1_ × Spring_T + *β*_2_ × V_Wind + *β*_3_ × Rain_S + *β*_4_ × Rain_L + *ε* (1)

where Y is the standardized detrended log-transformed RLR population; *β*_0_ is the constant intercept; *β*_1–4_ are the standardized partial regression coefficients; and *ε* is the random error term.

To address potential multicollinearity among meteorological predictors and quantify their individual importance, hierarchical partitioning was employed. This method calculates the independent contribution (*R*^2^) of each factor to the total variance explained. The direction of influence was determined using Pearson correlation coefficients (*r*).

## 3. Results

### 3.1. RLR Population Dynamics in Western Hunan

**Figure 2:**
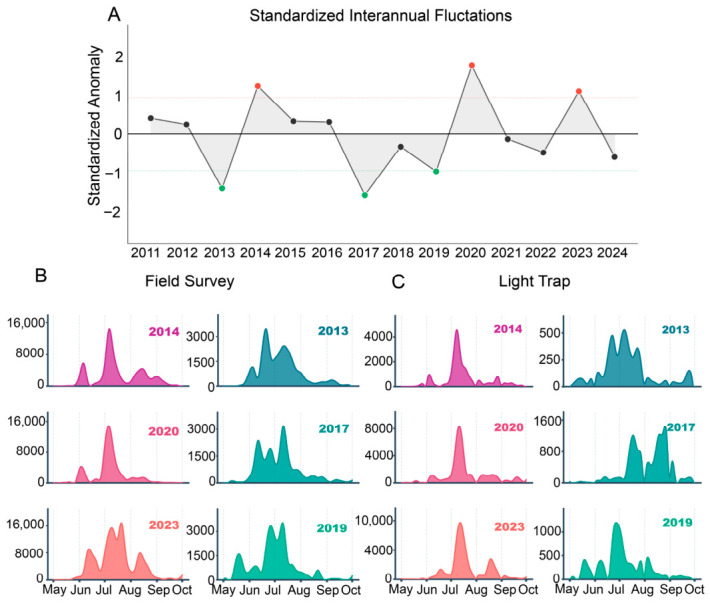
Interannual and seasonal RLR population variations in Western Hunan from 2011 to 2024. (**A**) Standardized interannual anomalies of RLR abundance in field surveys in July. Red dots denote years with heavy occurrence (abundance > 1 std above the mean), green dots denote years with light occurrence years (abundance < 1 std below the mean), and black dots denote other years. (**B**) The 5-day RLR abundance from field survey data during heavy occurrence years (2014, 2020, 2023; **left column**) and light occurrence years (2013, 2017, 2019; **right column**). (**C**) Daily RLR abundance from light trap data during heavy occurrence years and light occurrence years. The curves in (**B**,**C**) are derived from a spline-smoothing process based on daily or five-day RLR counts.

Based on 14 years of monitoring (2011–2024), we identified significant interannual variations in RLR abundance during July in Western Hunan (Figure 2A). Over this period, RLR abundance peaked in 2020, as an exceptional outbreak year, and reached a minimum observed in 2017. Such interannual fluctuations—notably the 2020 surge and 2024 crash—underscore the episodic nature of the primary infestation period. To explore the potential drivers of this interannual variability, three heavy occurrence years and three light occurrence years were selected based on standard deviation (std) criteria for subsequent comparative and composite analyses. Specifically, years with an abundance exceeding 1 std above the mean were identified as heavy infestation years, while those falling more than 1 std below the mean were classified as light years.

Seasonal patterns of RLR abundance derived from field surveys (moths occurred in rice paddies) revealed three main peaks throughout the entire year, with the most prominent and highest-magnitude peak consistently occurring in July (Figure 2B). RLR abundance derived from light traps (consists of recent immigrants and locally emerging moths and is more sensitive to immigration) exhibits multiple peaks but consistently shows the highest peak around July (Figure 2C). The first appearance of RLR occurred earlier in light-trap captures (late March) than in field surveys (early April). In heavy occurrence years (2014, 2020, and 2023), the July population is characterized by a sudden and massive arrival, forming a steep, unimodal peak, with field counts often exceeding 10,000 individuals—indicating concentrated landing of external migrants. In contrast, light occurrence years (2013, 2017, and 2019) exhibit a low-magnitude, dispersed pattern, with counts consistently below 3000 and lacking any dominant peak.

**Figure 3:**
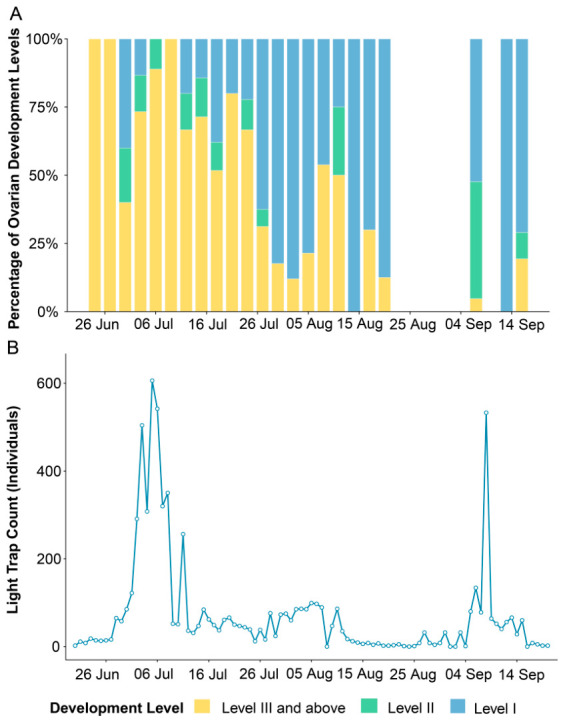
Population data and ovarian development of RLR in Zhijiang County in 2024. (**A**) Percentage of ovarian development stages assessed by dissection. (**B**) Daily light-trap catches.

A case study of ovarian dissection data from Zhijiang County revealed distinct stages in the RLR’s lifecycle and its migration patterns (Figure 3). During the initial light-trap peak (30 June to 9 July 2024), among all 81 collected female moths, 7.4% (6/81) exhibited Level 1 ovaries, 84.0% (68/81) reached Level 3 or beyond, and the mating rate is 90.1% (73/81) (Supplementary Table S1). This period coincided with the rice tillering stage (Supplementary Figure S6). Subsequently, from July 27 to August 8, a total of 110 females were examined, of which 77.3% (85/110) exhibited Level 1 ovaries, 21.8% (24/110) exhibited Level 3 or beyond, and the mating rate decreased to 23.6% (26/110). During this period, the rice crop transitioned into the booting stage. Between September 5 and September 16, despite a diminished population density—of which only 55 females were available for dissection—65.5% (36/55) of the individuals still maintained Level 1 ovaries, 12.7% (7/55) exhibited Level 3 or beyond, and the mating rate further declined to 21.8% (12/55). By this stage, the rice had reached maturity, with harvesting largely underway.

### 3.2. Main Source Areas for RLR Immigration to Western Hunan

**Figure 4:**
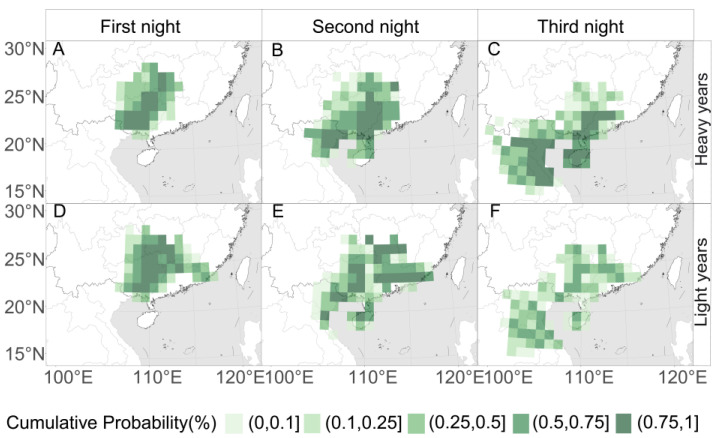
Comparison of potential source areas of RLR for Western Hunan between (**A**–**C**) heavy occurrence years and (**D**–**F**) light occurrence years during 2011–2024. The maps are based on the cumulative probability distribution of backward trajectory endpoints for the (**A**,**D**) first, (**B**,**E**) second, and (**C**,**F**) third nights prior to arrival.

Backward trajectory simulations of the July migration peaks reveal that key source regions for RLR in Western Hunan during the primary infestation period generally consist of Guangxi, Hainan, Laos, and Vietnam (Figure 4). Among these, Guangxi serves as the most critical direct source (first and second nights; Figure 4A,B,D,E), while overseas sources from Laos and Vietnam provide essential long-distance contributions (third night; Figure 4C,F). Overall, RLR during this period originates mainly from areas southwest of Western Hunan, whereas heavy and light occurrence years differ markedly in spatial concentration and trajectory evolution.

In heavy occurrence years, the backward trajectory endpoints show a high degree of spatial concentration. On the first night, source areas are concentrated in southeastern Guizhou and Guangxi, representing the direct nearby source bases. On the second night, sources are primarily centered in central Guangxi and northern Laos. On the third night, trajectories extend further back to Laos, north-central Vietnam, Hainan, and the southern coastal regions of China. This stepped distribution of high-probability areas establishes a stable “aerial corridor” extending from the northern Indochinese Peninsula through South China into Western Hunan. In contrast, source areas in light occurrence years are characterized by fragmentation and dispersion. Although the first-night sources resemble those of heavy occurrence years, second-night and third-night sources become scattered across South China, Laos, and central Vietnam, with no coherent migration pathway.

### 3.3. Meteorological Drivers of Interannual Variability in RLR Populations

**Figure 5:**
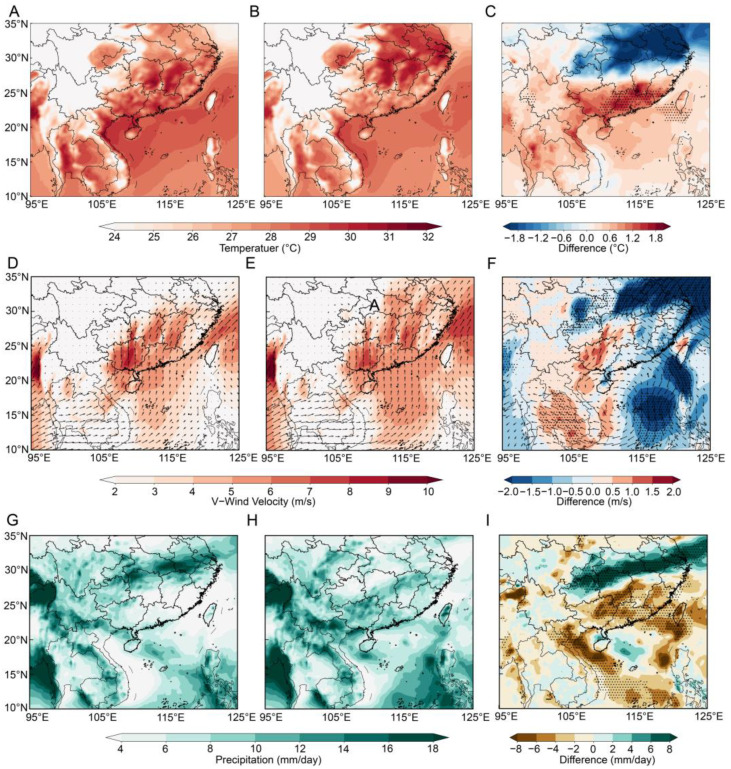
Comparison of meteorological factors between heavy occurrence years and light occurrence years during the peak migration period of RLR in July for Western Hunan. (**A**–**C**) Mean temperature in May (units: °C); (**D**–**F**) V-wind velocity (units: m/s) and wind vectors (arrows) in early and mid-July (1–20 July); (**G**–**I**) Mean daily precipitation in early and mid-July (1–20 July; units: mm/day). (**A**,**D**,**G**) Climatological fields for heavy occurrence years, (**B**,**E**,**H**) for light occurrence years, and (**C**,**F**,**I**) the differences between heavy and light occurrence years (heavy minus light). Dotted areas indicate differences that are statistically significant at the 95% confidence level based on *t*-test.

To further elucidate the climatic driving mechanisms underlying the differences in immigration levels between high and light occurrence years, the key meteorological fields were compared between the two types of years (Figure 5).

First, spring temperature anomalies established a thermodynamic foundation for large initial population accumulation. Source regions identified by backward trajectory analysis, namely the Indochinese Peninsula and southern South China, are significantly warmer during May in heavy occurrence years (Figure 5A–C). The difference field reveals a distinct positive temperature anomaly (0.6~1.8 °C) that precisely overlaps with the primary source areas. Second, the efficiency of atmospheric transport dynamics determined the scale of immigration. In heavy occurrence years, the southerly jet stream crossing Guangxi was substantially stronger (Figure 5D–F), with strong meridional wind zones (>8 m/s) extending significantly further north into Western Hunan compared to light occurrence years. The difference field indicates a positive deviation of 0.5–2.0 m/s along the primary migration pathway. Finally, the spatial pattern of the precipitation field formed an ideal outbreak mechanism. In contrast to the relatively uniform and moderate rainfall observed in light occurrence years, heavy occurrence years were characterized by a pronounced northward shift in the rain belt (Figure 5G–I). The difference field exhibits significant positive precipitation anomalies (4–8 mm/day) over the middle and lower reaches of the Yangtze River and significant negative precipitation anomalies over the Indochinese Peninsula and parts of southern China.

### 3.4. Multiple Linear Regression Model for Interannual Variability in RLR Populations

**Figure 6:**
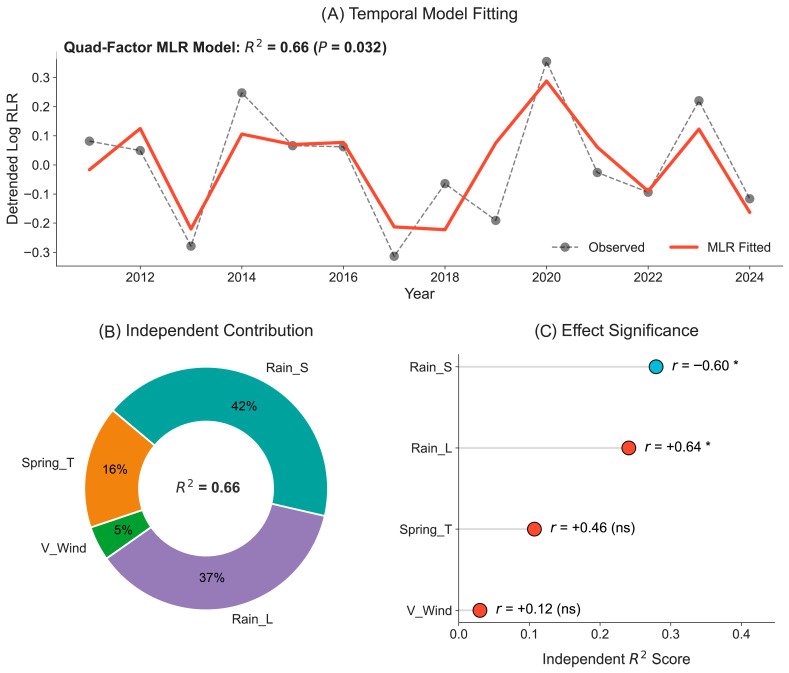
Model fitting and quantitative assessment of key meteorological factors influencing July RLR abundance in Western Hunan. (**A**) Field survey (dashed gray line) and MLR model-fitted curves (solid red line; Equation (1)) RLR abundance (*R*^2^ = 0.66, *p* = 0.032). (**B**) Independent contribution of each factor to the total variance explained (*R*^2^ = 0.66), estimated using hierarchical partitioning. (**C**) Effect significance and importance of meteorological factors. The x-axis shows the independent *R*^2^ score, and labels provide Pearson’s correlation coefficient (*r*) for each factor. “*” and “ns” denote statistical significance (*p* < 0.05) and non-significance (*p* > 0.05), respectively. Blue and red dots represent negative and positive correlations, respectively.

Spatial correlation analysis further reveals a close relationship between interannual variations of RLR abundance and those of meteorological factors over the past 14 years (Supplementary Figure S4). To quantify the synergistic effects of these meteorological drivers, a quad-factor multiple linear regression (MLR) model was constructed (Equation (1)). The model explains 66.1% of the interannual variance in RLR abundance in Western Hunan (*R*^2^ = 0.66, *p* = 0.032; Figure 6A). Regression diagnostics were conducted to ensure the robustness of the MLR model (Supplementary Figure S5). The fitted curve accurately replicates the fluctuations of the RLR population from 2011 to 2024, successfully capturing heavy occurrence years (e.g., 2014, 2020, and 2023) and light occurrence years (e.g., 2013, 2017). This high degree of temporal consistency underscores the model’s potential as a predictive tool for regional RLR risk assessment.

Hierarchical partitioning analysis suggests a prominent role for precipitation patterns in governing RLR population fluctuations (Figure 6B). Precipitation-related factors cumulatively account for approximately 80% of the total explained variance, with source-region precipitation (Rain_S, 42%) and local precipitation (Rain_L, 37%) appearing as the top contributors. In contrast, the relative contributions of thermodynamic factors (Spring_T, 16%) and transport dynamics (V_Wind, 5%) are considerably lower. It is worth noting that, given the limited sample size, these percentage estimates should be interpreted cautiously as indicators of relative importance rather than precise absolute contributions.

The effect significance and directionality (Figure 6C) further clarify the role of each factor. Local precipitation (Rain_L) exhibits a significant positive correlation (*r* = 0.64, *p* < 0.05), acting as the primary driver for forced RLR landing. Conversely, source-region precipitation (Rain_S) shows a significant negative correlation (*r* = −0.60, *p* < 0.05), statistically confirming that reduced rainfall (dry conditions) in early and mid-July favors the initial takeoff and mass emigration from source areas. Although Spring_T (*r* = 0.46) and V_Wind (*r* = 0.12) show positive weights, aligning with the “warm-source” and “aerial-corridor” hypotheses, their individual effects within this linear framework did not reach statistical significance (*p* > 0.05).

## 4. Discussion

Our 14-year systematic analysis reveals substantial interannual variability in RLR abundance in Western Hunan. The frequent abrupt transitions between high- and low-abundance years highlight the episodic and unstable nature of regional infestations of RLR. Thus, it is necessary to clarify the characteristics of RLR occurrence, figure out its source areas and migration patterns, and determine possible drivers of these interannual fluctuations.

Seasonally, field survey data revealed three activity peaks of RLR in Western Hunan throughout the year, corresponding to the third, fourth, and fifth generations in this region, which cause severe damage (Supplementary Figure S6). The ovarian dissection results indicate that the first peak (early July) was characterized by large-scale immigration of exogenous populations, as evidenced by the high proportion of females with Level 3+ ovaries and a high mating rate. The second peak (late July to early August) showed a dominance of Level 1 ovaries and a low mating rate, suggesting local reproduction and subsequent emigration. The third peak (early September) exhibited low moth abundance and a high proportion of Level 1 ovaries under unfavorable rice conditions, reflecting the return migration of external populations. Overall, the early June and mid-July peaks represent successive waves of massive immigration, the early-to-mid August peak corresponds to local reproduction and emigration, and the early September peak marks return migration. Among these, the mid-July peak is considered the primary infestation period, with the highest population abundance and longest damage duration.

These population dynamics and the associated RLR developing stages are consistently influenced by local rice cultivation systems. In Western Hunan, where single-cropping middle rice predominates (late May to mid-September), the first migration peak occurs in early June, representing northward immigration that settles on local rice at the tillering stage, with initial RLR detections by light traps as early as April. Later in the season, although late-season maturation and harvest in late August limit the further growth of field populations, light trap monitoring continues to detect a potential fourth peak during the post-harvest period. This phenomenon reflects the southward return migration of the insect pests. In contrast, eastern and southern Hunan feature double-cropping systems (early rice from early April to mid-July and middle rice from late June to late October), where the first migration peak typically appears in early to mid-May [20,29].

The profound influence of cropping systems on migratory pest dynamics is further exemplified by another migratory rice pest—the brown planthopper (BPH): the long-term prevalence of double-cropping in southern China supplies abundant host resources for both immigration and return migration, whereas the expansion of single-season mid-rice at the expense of double-cropping in the middle-lower Yangtze River valley has likely increased return-migration populations [30]. Such shifts not only extend suitable feeding periods but also ensure continuous host availability through spatiotemporal synchronization with double-cropping systems in southern China, thereby elevating regional outbreak risks. Although specific research on RLR remains limited, similar impacts are highly probable, given its comparable migratory trajectories and dependence on host phenology, which is supported by our results.

For interannual variations, standardized anomalies identified three distinct outbreak years (2014, 2020, 2023) and three light occurrence years (2013, 2017, 2019); these years formed the basis for subsequent comparative analyses between heavy and light occurrence years. A comparative analysis between heavy and light occurrence years suggests that interannual differences in July abundance are fundamentally driven by the intensity and concentration of immigrant numbers. In heavy occurrence years, the sudden and massive arrival of external migrants—indicated by the steep, unimodal peak with counts exceeding 10,000—suggests concentrated landing. In light occurrence years, the low-magnitude, dispersed pattern reflects the absence of such massive immigration. This systematic difference demonstrates that the severity of July infestations in Western Hunan is primarily determined by the magnitude of external immigration rather than by local population growth.

Back-trajectory analysis identified Guangxi, Hainan, Laos, and Vietnam as the primary source regions of RLR immigrating into Western Hunan during July. The concentrated backtracking endpoints confirm a stable “aerial corridor” extending from the northern Indochinese Peninsula through South China into Western Hunan in heavy occurrence years compared with light occurrence years. The meteorological conditions also confirm the “aerial corridor” assumption, i.e., the southerly jet stream was remarkably more robust, with high V-component zones (>8 m/s) extending significantly across Guangxi north into Western Hunan in high occurrence years, which facilitates more efficient large-scale long-distance northward transport. In addition, May temperature distribution in source regions effectively shortened the developmental duration of the RLR and accelerated population growth, thereby providing a massive initial population base for the northward migration in July, dry conditions in source areas favoring takeoff, strong winds in transit facilitating flight, and heavy rain at the destination inducing forced settling. These factors together cause a heavy occurrence in Western Hunan. This configuration represents a critical meteorological driver of the massive RLR outbreaks in Western Hunan. The influence of wind and rainfall on the migration of RLR populations has also been reported in earlier case studies [7,8,9,28].

We developed a linear model to quantify the contributions of meteorological drivers and to evaluate the relative importance of each factor. The model demonstrated high statistical reliability in capturing the interannual fluctuations of RLR populations, confirming that meteorological conditions largely determine the severity of RLR outbreaks in Western Hunan. These results point toward a scenario where temperature and wind establish the necessary conditions for migration, while precipitation patterns likely act as a decisive factor in determining the actual scale and localization of RLR infestation. Together, these results confirm the “Dry-Takeoff, Wet-Settlement” mechanism. Among all factors, precipitation-related factors collectively account for nearly 80% of the independent contribution, with source-region precipitation and local precipitation serving as two primary determinants. These results suggest that while temperature and wind establish the potential for migration, precipitation patterns ultimately dictate the scale and localization of RLR occurrence.

Although this study systematically analyzed the RLR population dynamics and their meteorological drivers in Western Hunan, several limitations should be acknowledged. On the one hand, pest monitoring data relied primarily on light traps and field surveys, and the limited spatial coverage of monitoring sites may not fully capture the spatiotemporal heterogeneity of pest occurrence. Future studies should integrate automatic monitoring devices, radar monitoring, and other complementary technologies to enhance spatial representativeness and data continuity. On the other hand, the impact of agricultural management practices (such as cropping systems, varietal resistance, fertilization, and pesticide application) was not explicitly considered. Incorporating agricultural remote sensing data and crop statistics into multi-factor modeling frameworks would help improve the explanatory power of pest dynamics.

In summary, this study clarified the occurrence patterns of RLR in Western Hunan, identified major source regions through trajectory analysis, and highlighted the key meteorological factors influencing RLR population dynamics through composite analysis and modeling. These findings provide valuable theoretical and practical insights for precise monitoring and source-based control of RLR in the region.

## Data Availability

The raw data supporting the conclusions of this article will be made available by the corresponding author on request.

## Supplementary Materials

The following supporting information can be downloaded at: https://www.mdpi.com/article/10.3390/insects17050466/s1, Table S1: Ovarian dissection data of the RLR in Zhijiang, Western Hunan, 2024. Figure S1: Climatological averaged monthly precipitation and near surface air temperature (units: 2 m) in western Hunan from 2011 to 2024. Figure S2: Wind rose analysis of western Hunan during June and July from 2011 to 2024. Figure S3: Map showing the definition of regions for calculating key meteorological variables. Figure S4: Spatial correlations between metrological field and RLR population in July. Figure S5: Statistical assessment of the MLR model robustness. Figure S6: Schematic diagram of seasonal RLR dynamics in relation to rice phenology in Hunan province. Reference [31] are cited in the supplementary materials.
