# Supplementary material for: Migration Patterns and Meteorological Drivers of the Rice Leaf Roller in Western Hunan Province, China
Source: Insects. 2026 Apr 30;17(5):466. doi: 10.3390/insects17050466 (PMC13207645; doi:10.3390/insects17050466)
Supplement: Supplementary file 1 [file insects-17-00466-s001.zip › insects-4250952-supplementary.pdf]

Supplementary Material

Migration Patterns and Meteorological Drivers of the Rice Leaf Roller in Western Hunan Province, China

Jia-Hao Zhang [1], Xue-Yan Zhang [1], Yi-Yang Zhang [2], Jian Tian [3], Xiao-Yu Ouyang [1], Li Yin [4], Yan Wu [5], Juan Zeng [2], Shi-Yan Zhang [1,*] and Gao Hu [1,5]

[1] State Key Laboratory of Agricultural and Forestry Biosecurity, College of Plant Protection, Nanjing Agricultural University, Nanjing 210095, China
[2] National Agro-Tech Extension and Service Center, Ministry of Agriculture and Rural Affairs of the People's Republic of China, Beijing 100026, China
[3] Plant Protection and Plant Quarantine Station of Zhijiang Dong Autonomous County, Huaihua 418400, China
[4] Hunan Plant Protection and Plant Quarantine Station, Changsha 411229, China
[5] Guizhou Key Laboratory of Agricultural Biosecurity, Guiyang University, Guiyang 550005, China
* Correspondence: zhangsy@njau.edu.cn

Contents of this file

Table S1

Figures S1-S6

References

Table S1. Ovarian dissection data of the RLR in Zhijiang, Western Hunan, 2024.

Date (MM/DD)	Number of individuals dissected	Number of individuals with spermatophores	Level 1	Level 2	Level 3	Level 4	Level 5
6/24	1	1	0	0	1	0	0
6/27	1	1	0	0	0	1	0
6/30	10	9	4	2	2	2	0
7/3	16	16	2	2	8	3	0
7/6	27	26	0	3	11	6	7
7/9	28	28	0	0	8	9	11
7/12	15	12	3	2	5	3	2
7/15	21	17	3	3	14	0	1
7/18	29	28	11	3	6	6	3
7/21	5	5	1	0	0	0	4
7/24	14	12	2	1	0	2	4
7/27	16	5	10	1	2	0	3
7/30	17	3	14	0	0	2	1
8/2	50	8	44	0	2	0	4
8/5	14	3	11	0	1	2	0
8/8	13	7	6	0	7	0	0
8/11	4	3	1	1	1	1	0
8/14	1	0	1	0	0	0	0
8/17	10	3	7	0	1	0	2
8/20	16	2	14	0	1	0	1
8/23	0	0	0	0	0	0	0
8/26	0	0	0	0	0	0	0
8/29	0	0	0	0	0	0	0
9/1	0	0	0	0	0	0	0
9/4	0	0	0	0	0	0	0
9/7	21	5	11	9	1	0	0
9/10	0	0	0	0	0	0	0
9/13	3	0	3	0	0	0	0
9/16	31	7	22	3	6	0	0

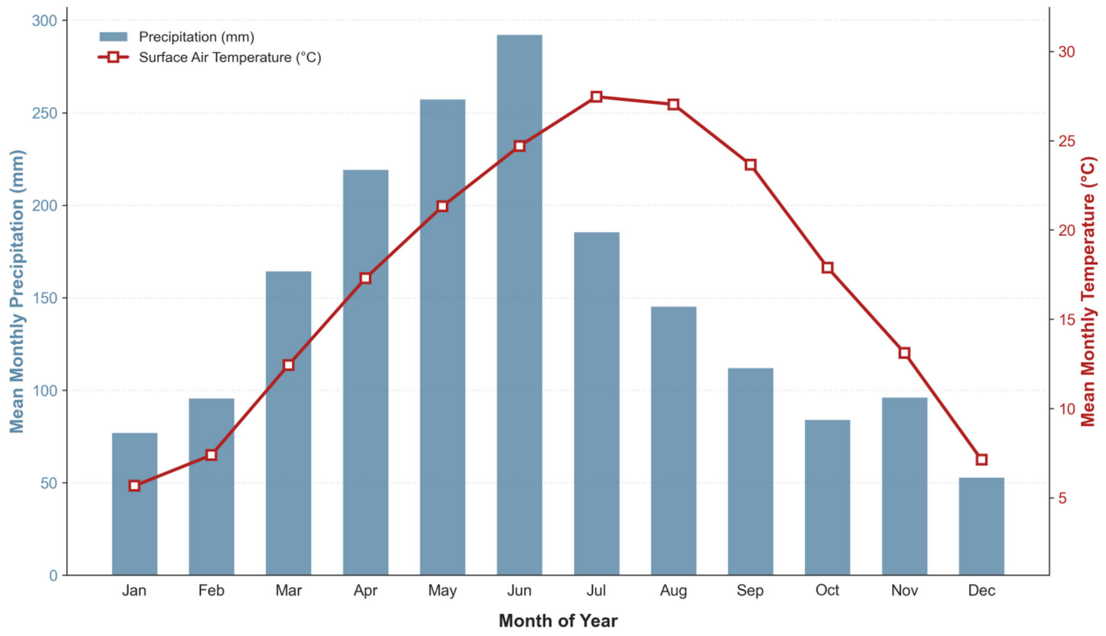

Figure S1. Climatological averaged monthly precipitation and near surface air temperature (units: 2 m) in western Hunan from 2011 to 2024. Blue bars represent the mean monthly precipitation (mm), and the red line with markers denotes the mean monthly near surface air temperature (°C).

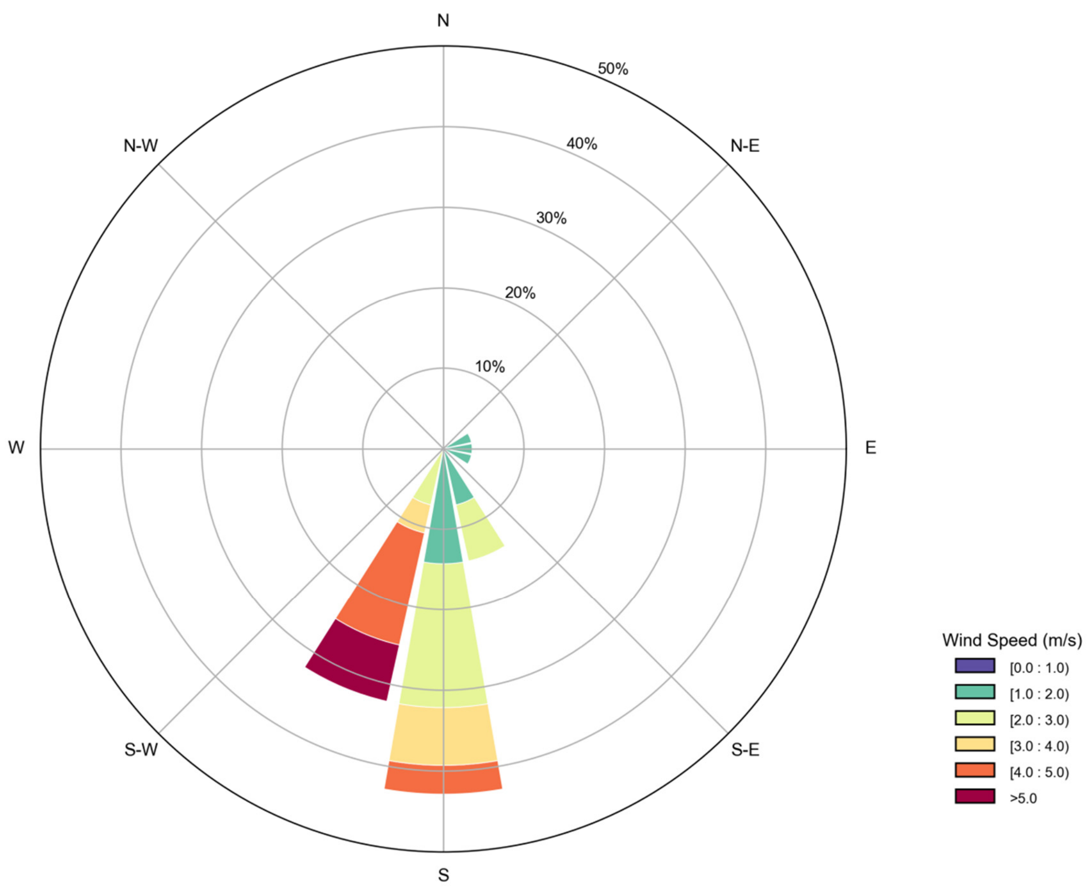

Figure S2. Wind rose analysis of western Hunan during June and July from 2011 to 2024. The plot illustrates the distribution of prevailing wind directions and speeds. Different colors represent wind speed intervals (units: m/s), and the radial distance indicates the frequency occurrence of winds of each direction.

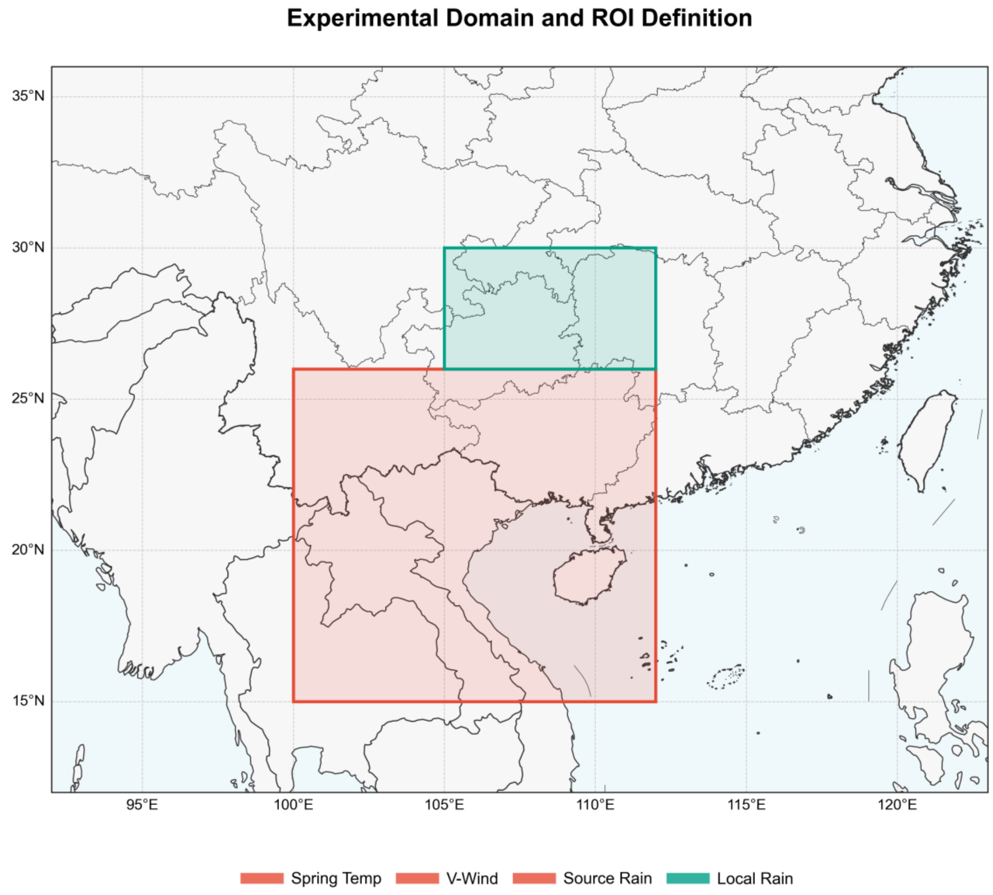

Figure S3. Map showing the definition of regions for calculating key meteorological variables. The green box represents the area used for the local precipitation index (Rain_L); the red rectangle denotes the area for spring near-surface air temperature (Spring_T), meridional wind (V_Wind), and source region precipitation (Rain_S).

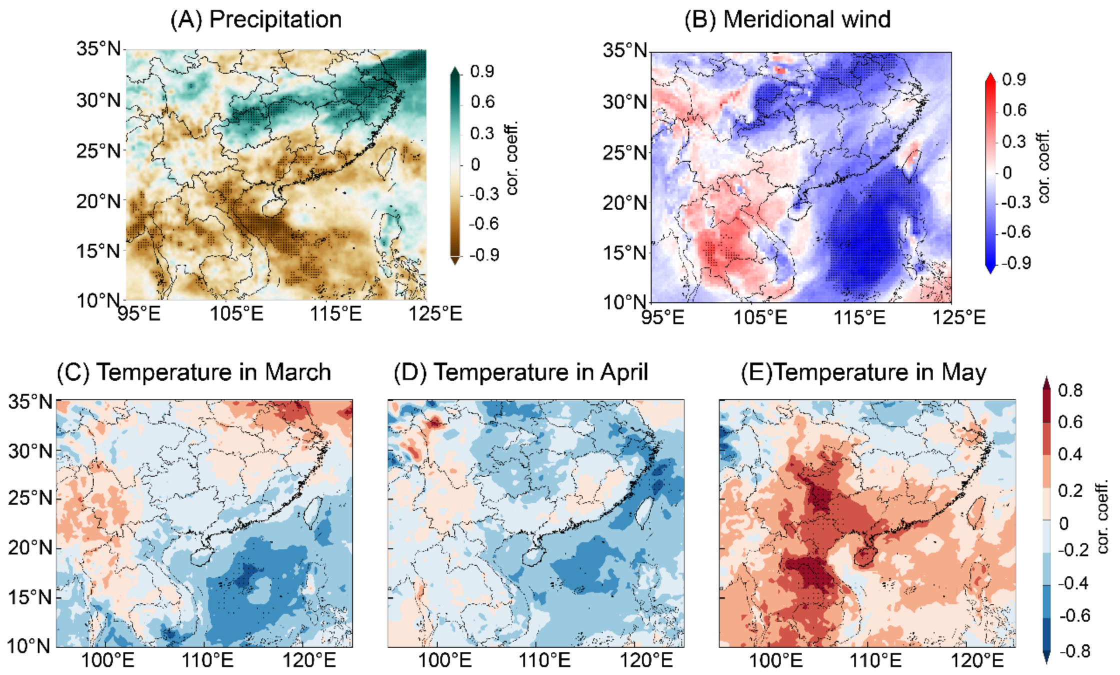

Figure S4. Spatial correlations between metrological field and RLR population in July. (A) precipitation, (B) meridional wind, and (C-E) monthly mean spring temperatures (March, April, and May). Color shading represents the Spearman correlation coefficient (r). Stippled areas denote regions where r is statistically significant at the 95% confidence level (*p<0.05*). All data were detrended prior to analysis to remove long-term linear trends.

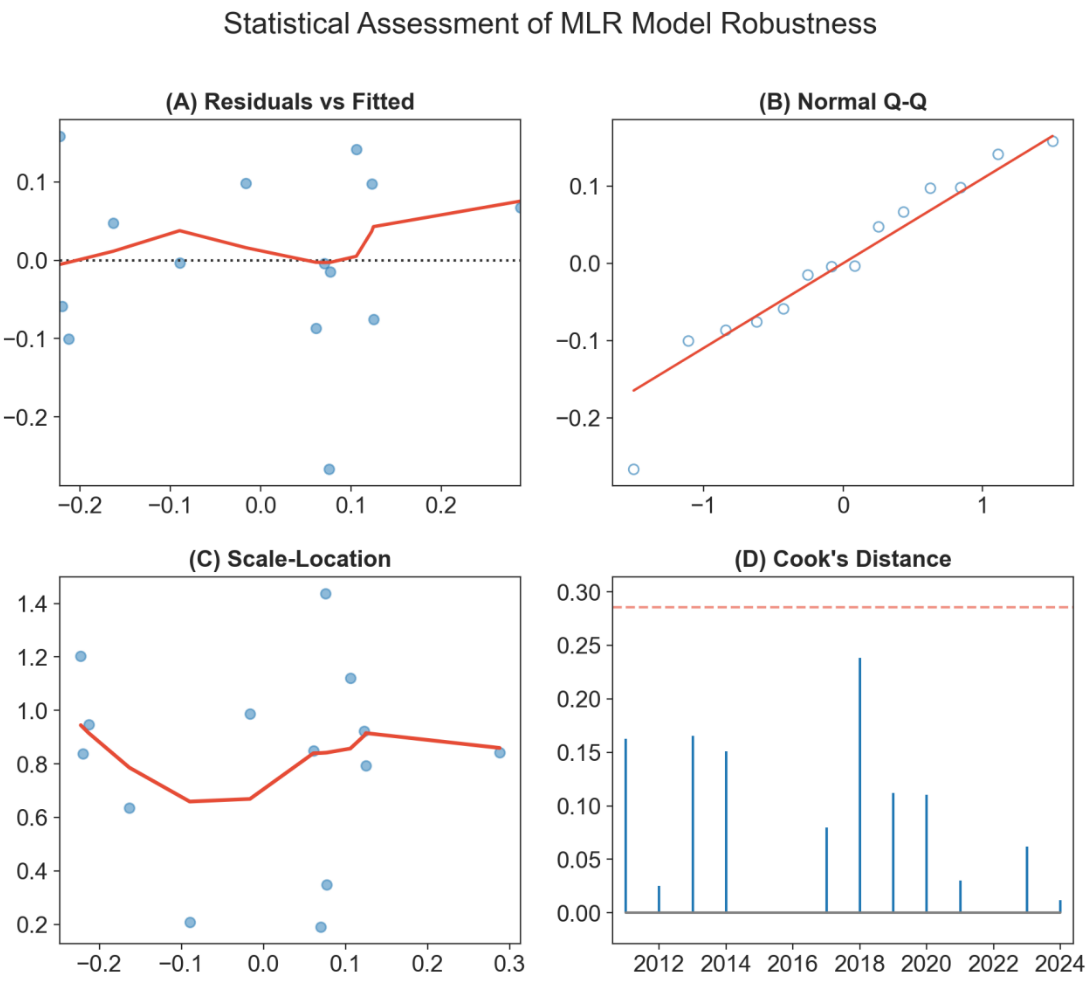

Figure S5. Statistical assessment of the MLR model robustness. (A) Residuals vs Fitted plot. Residuals are randomly scattered around the zero line with a nearly horizontal LOWESS trend. (B) Normal Q-Q plot. The close alignment of data points with the diagonal reference line confirms the normality of residuals. (C) Scale-Location plot. The relatively stable spread of standardized residuals indicates no significant heteroscedasticity. (D) Cook's Distance plot. All values remain well below the pre-defined threshold (dashed red line), confirming the absence of highly influential outliers that could bias the regression coefficients. Furthermore, the Variance Inflation Factor (VIF) for all predictors ranged from 1.28 to 1.78, indicating no multicollinearity. The Durbin-Watson statistic (2.966) indicates some negative serial correlation, which is within a typical range for ecological time-series data and does not compromise the statistical significance or the interpretation of the key predictors. Collectively, these diagnostics demonstrate that the model satisfies the fundamental Gauss-Markov assumptions, ensuring the statistical integrity and reliability of the analytical results.

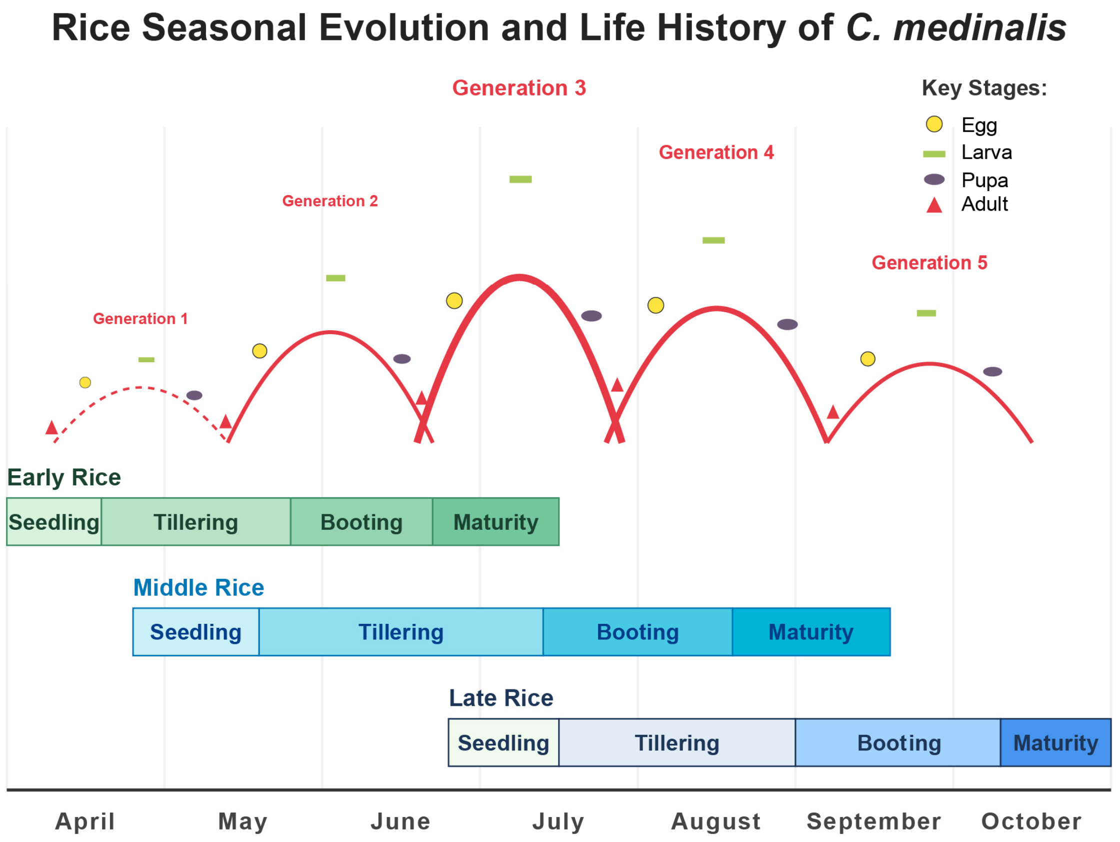

Figure S6. Schematic diagram of seasonal RLR dynamics in relation to rice phenology in Hunan province. The horizontal bars delineate key rice phenological stages—Seedling, Tillering, Booting, and Maturity—for early, middle and late rice across the production season from April to October. The red lines depict RLR population dynamics: dashed lines represent immigration and emigration phases; solid lines represent local generational expansion. Line thickness qualitatively indicates relative population size, with thicker lines denoting larger populations. The peak population is typically coinciding with the late stages of early rice and the early stages of middle/late rice (in July). Colored markers indicate the approximate RLR development stages. Middle-season rice is predominant in Western Hunan, while early and late-season rice are grown in other parts of Hunan. Information is derived from [1-4].

References

1. Zhu, X.X.; Zhang, Z.B.; Yin, L.; et al. Analysis of the occurrence characteristics and trends of rice pests in Hunan Province. *Hunan Agricultural Sciences*, 2012, 23, 74–77.
2. Zhu, X.X.; Tan, X.P.; Wang, B. Occurrence patterns and control strategies of the "two migration" pests of rice in Hunan Province. *Chinese Journal of Plant Protection*, 2016, 36, 27–31.
3. Deng, W.; Tan, J.Y.; Liu, B.; et al. Study on the distribution of major cultivated rice varieties in Hunan Province. *Hunan Agricultural Sciences*, 2019, 03, 85–88.
4. Zhou, X.J.; Zou, D.S. Reform and development of paddy field cropping system in Hunan Province. *Tillage and Cultivation*, 2004, 02, 1–2.
